# Global Advances in Tomato Virome Research: Current Status and the Impact of High-Throughput Sequencing

**DOI:** 10.3389/fmicb.2021.671925

**Published:** 2021-05-21

**Authors:** Mark Paul Selda Rivarez, Ana Vučurović, Nataša Mehle, Maja Ravnikar, Denis Kutnjak

**Affiliations:** ^1^Department of Biotechnology and Systems Biology, National Institute of Biology, Ljubljana, Slovenia; ^2^Jožef Stefan International Postgraduate School, Ljubljana, Slovenia; ^3^Faculty of Agriculture, University of Belgrade, Belgrade, Serbia; ^4^School for Viticulture and Enology, University of Nova Gorica, Nova Gorica, Slovenia

**Keywords:** tomato, virome, high-throughput sequencing, metagenomics, virus diversity, virus discovery, virus ecology, virus epidemiology

## Abstract

Viruses cause a big fraction of economically important diseases in major crops, including tomato. In the past decade (2011–2020), many emerging or re-emerging tomato-infecting viruses were reported worldwide. In this period, 45 novel viral species were identified in tomato, 14 of which were discovered using high-throughput sequencing (HTS). In this review, we first discuss the role of HTS in these discoveries and its general impact on tomato virome research. We observed that the rate of tomato virus discovery is accelerating in the past few years due to the use of HTS. However, the extent of the post-discovery characterization of viruses is lagging behind and is greater for economically devastating viruses, such as the recently emerged tomato brown rugose fruit virus. Moreover, many known viruses still cause significant economic damages to tomato production. The review of databases and literature revealed at least 312 virus, satellite virus, or viroid species (in 22 families and 39 genera) associated with tomato, which is likely the highest number recorded for any plant. Among those, here, we summarize the current knowledge on the biology, global distribution, and epidemiology of the most important species. Increasing knowledge on tomato virome and employment of HTS to also study viromes of surrounding wild plants and environmental samples are bringing new insights into the understanding of epidemiology and ecology of tomato-infecting viruses and can, in the future, facilitate virus disease forecasting and prevention of virus disease outbreaks in tomato.

## Introduction

Tomato (*Solanum lycopersicum* L.) is one of the most economically valuable fruit or vegetable crops worldwide, valued at 93.9 billion US dollars in 2018, with yield estimated at 180.8 million tons in 2019 (FAOSTAT, 2020). Tomato production is affected by numerous diseases and, among them, viruses are considered an important production-limiting factor (Hanssen et al., 2010b; Souiri et al., 2019; Hančinský et al., 2020; Ong et al., 2020). It was estimated that almost half of the emerging crop diseases can be attributed to plant viruses (Anderson et al., 2004), which could amount up to around a quarter of the overall attainable yields in major crops, including tomato (Oerke, 2006). Worldwide economic damages caused by viruses in crops are difficult to estimate; however, rough approximations indicate yield losses range from 30 to 50 billion US dollars annually (Sastry, 2013). Yield and economic losses due to virus diseases in tomato vary greatly and are often dependent on the virus species and growing region.

Until a decade ago, viruses were mostly analyzed using targeted detection techniques such as ELISA, PCR, and Sanger sequencing (Katsarou et al., 2019). These methods are widely available in molecular biology labs, but they require *a priori* information on viral genomes or serological properties of viral species that might be present in a sample. The study of a whole community of viruses (i.e., viromes), including unknown ones, became possible due to the rapid decrease in high-throughput sequencing (HTS) costs (NIH-NHGRI, 2019) and the availability of the improved data analysis tools (Villamor et al., 2019). This circumvented challenges of targeted detection of plant viruses and contributed useful ecological and epidemiological insights. First examples of the use of HTS for detection of plant viruses are now over a decade old (Adams et al., 2009a; Al Rwahnih et al., 2009; Donaire et al., 2009; Kreuze et al., 2009). Currently, HTS is used for discovery of many novel viruses and is now establishing its position as one of the classical approaches in plant virology research and diagnostics laboratories (Massart et al., 2019). HTS is often employed with one of the possible nucleic acid preparation approaches, such as sequencing of double-stranded (ds)RNA, small (s)RNAs, total (tot)DNA after rolling circle amplification, virion-associated RNA (VANA), and total (tot)RNA after ribosomal (r)RNA depletion (Pecman et al., 2017; Ma et al., 2019; de Nazaré Almeida dos Reis et al., 2020; Gaafar and Ziebell, 2020). Each method has its pros and cons (Roossinck, 2017) and should be selected according to the aims of the study; e.g., sequencing rRNA depleted totRNA or sRNA might be the most straightforward and generic approaches for detection of a wide range of viruses in single samples or relatively small sample pools (Pecman et al., 2017). On the other hand, dsRNA or VANA might be beneficial, when trying to enrich for viruses in, e.g., large pools of starting plant material (Ma et al., 2019).

Both classical (Sanger) sequencing and HTS are regularly used for discovery of new viruses in tomato; however, the usage of the latter is evidently increasing in the past few years. In the first part of this review, we discuss the discovery of 45 novel virus species in tomato within the 2011–2020 period and contextualize the role of HTS in these findings. Moreover, a post-discovery characterization of new viruses represents an important step toward understanding their biological and/or economical relevance (Massart et al., 2017; Hou et al., 2020); thus, we also systematically reviewed to which extent such characterization have been performed for newly discovered viruses associated with tomato. Beyond discovering and detecting viruses in tomato, HTS can enable a broader look into the virome of tomato on a defined geographical scale, a virome of surrounding plants and vectors, and possible exchanges among those communities. HTS-based virome studies of tomato and surrounding wild plant species (which can serve as reservoirs for viruses) (Hančinský et al., 2020; Ma et al., 2020), and environmental samples, such as water (which might serve as transmission pathway) (Bačnik et al., 2020), can also bring important insights into the understanding of the epidemiology of some tomato viruses.

Review of the past and recent discoveries of viruses in tomato shows that tomato is currently associated with at least 312 different viral species, which is likely, according to our knowledge, the largest recorded number known for any cultivated plant. Among those, many known and several recently discovered viruses cause significant economic damage in tomato production in different parts of the world. In the second part of this review, we focus on important tomato viruses, which caused significant economic losses in tomato production in the past decade and new virus discoveries in tomato, for which limited or no knowledge about potential impact on tomato health is available.

## HTS Has Become an Important Tool in Tomato Virus Discovery and Epidemiology Studies

**FIGURE 1:**
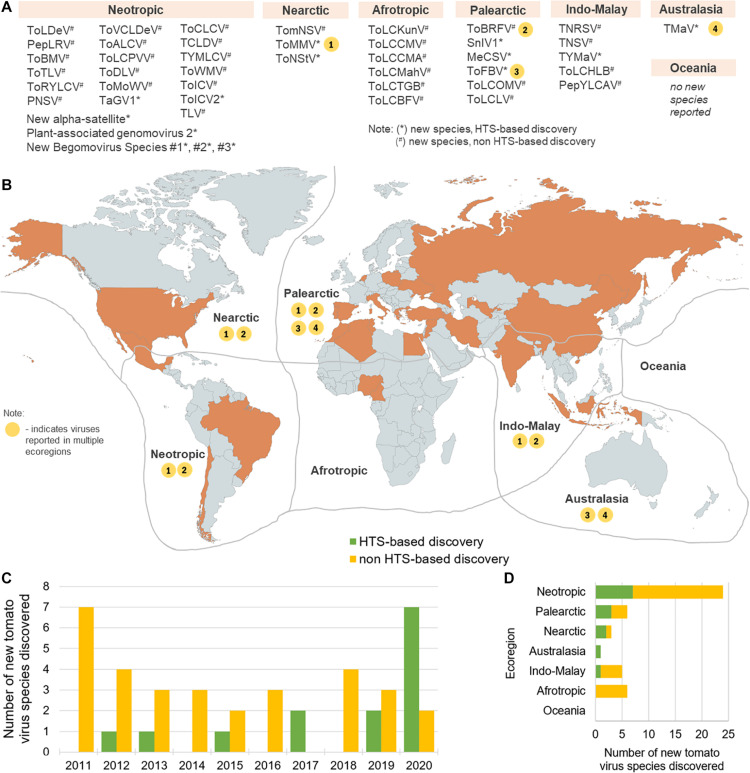
Global distribution of virus species newly discovered in the 2011 to 2020 period. Abbreviated virus names (see Table 1) are shown in groups corresponding to the ecozones in which they were discovered (Antarctic is not shown, for simplicity, China and Indonesia are included in Indo-Malay, and Mexico is included in the Neotropic ecoregion) **(A)**. In the map **(B)**, ecoregions are delimited by gray lines and countries colored in orange are top producers of tomato in the 2008–2018 period, recording at least 8.14 million tons of total tomato produced (FAOSTAT, 2020). Viruses detected in more than one ecozone are shown on the map as number-coded yellow circles (1–4) corresponding to the virus species designated in the list in **(A)**. The numbers of annual new tomato virus discoveries in the 2011–2020 period are shown in **(C)** and the numbers of new tomato virus discoveries per ecoregion in this time period are shown in **(D)**. The map was created using www.mapchart.net under the CC BY-SA 4.0 license.

Forty-five novel virus species were discovered in tomato in the recent decade (2011–2020) (Table 1). Majority of these discoveries were made in Neotropic and Palearctic countries and just in recent years (Figure 1). Out of this set, 14 were discovered using HTS, and in 2020 alone, seven novel species were discovered just by three HTS-based studies (Ciuffo et al., 2020; de Nazaré Almeida dos Reis et al., 2020; Ma et al., 2020). In this period, also four viral families were associated with tomato for the first time, in four HTS-based studies, i.e., *Iflaviridae* (Saqib et al., 2015), *Phenuiviridae* (Lecoq et al., 2019), *Kitaviridae* (Ciuffo et al., 2020), and *Genomoviridae* (de Nazaré Almeida dos Reis et al., 2020). Specifically, more than half of the RNA viruses were discovered using HTS (7/13), while most of the DNA viruses (including satellite virus species) were discovered using a non-HTS approach (24/32). Overall, non-HTS-based discovery approaches remain widely used for targeted, single species plant virus discoveries, while HTS has become a tool to discover multiple species, without *a priori* knowledge about possibly present viruses. In this section, the impact of HTS as an important tool in tomato viromics is further contextualized, from the discovery of viruses in individual plants, through the post-discovery characterization of new viruses, to the holistic analysis of plant viromes in agroecosystems.

**TABLE 1:** Novel virus species discovered from 2011 to 2020 and associated with tomato.

Family	Genus	Virus name	Acronym	Baltimore Group^1^	HTS-based discovery?	Sample and library preparation approach^2^/ Sequencing platform	Country(ies) where first reported	Publication
*Alphasatellitidae*	Unspecified	“New alphasatellite”	–	ssDNA	Yes	Total DNA-RCA/HiSeq 2500	Brazil	de Nazaré Almeida dos Reis et al., 2020
	*Colecusatellite*	Tomato leaf curl Cameroon alphasatellite	ToLCCMA	ssDNA	No	–	Cameroon	Leke et al., 2011
*Bromoviridae*	*Ilarvirus*	Solanum nigrum ilarvirus 1	SnIV1	(+)ssRNA	Yes	dsRNA/HiSeq 3000	France	Ma et al., 2020
		Tomato necrotic streak virus	TomNSV	(+)ssRNA	No	–	USA	Adkins et al., 2015
*Geminiviridae*	*Begomovirus*	“New begomovirus species #1”	–	ssDNA	Yes	Total DNA-RCA/HiSeq 2500	Brazil	de Nazaré Almeida dos Reis et al., 2020
		“New begomovirus species #2”	–	ssDNA	Yes	Total DNA-RCA/HiSeq 2500	Brazil	de Nazaré Almeida dos Reis et al., 2020
		“New begomovirus species #3”	–	ssDNA	Yes	Total DNA-RCA/HiSeq 2500	Brazil	de Nazaré Almeida dos Reis et al., 2020
		Pepper leafroll virus	PepLRV	ssDNA	No	–	Peru	Martínez-Ayala et al., 2014
		Pepper yellow leaf curl Aceh virus	PepYLCAV	ssDNA	No	–	Indonesia	Kesumawati et al., 2019
		Tomato apical leaf curl virus	ToALCV	ssDNA	No	–	Argentina	Vaghi Medina et al., 2018
		Tomato chlorotic leaf curl virus	ToCLCV	ssDNA	No	–	Brazil	Quadros et al., 2019
		Tomato chlorotic leaf distortion virus	TCLDV	ssDNA	No	–	Venezuela	Zambrano et al., 2011
		Tomato dwarf leaf virus	ToDLV	ssDNA	No	–	Argentina	Vaghi Medina and López Lambertini, 2012
		Tomato interveinal chlorosis virus	ToICV	ssDNA	No	–	Brazil	Albuquerque et al., 2012
		Tomato interveinal chlorosis virus-2	ToICV2	ssDNA	Yes	Total DNA-RCA/HiSeq 2000	Brazil	Rego-Machado et al., 2019
		Tomato latent virus	TLV	ssDNA	No	–	Cuba	Fuentes et al., 2016
		Tomato leaf curl Burkina Faso virus	ToLCBFV	ssDNA	No	-	Burkina Faso	Ouattara et al., 2017
		Tomato leaf curl Cameroon virus	ToLCCMV	ssDNA	No	–	Cameroon	Leke et al., 2011
		Tomato leaf curl Kunene virus	ToLCKunV	ssDNA	No	–	Namibia	Lett et al., 2020
		Tomato leaf curl Liwa virus	ToLCLV	ssDNA	No	–	Oman	Khan et al., 2014
		Tomato leaf curl Mahé virus	ToLCMahV	ssDNA	No	–	Seychelles	Scussel et al., 2018
		Tomato leaf curl Oman virus	ToLCOMV	ssDNA	No	–	Oman	Idris et al., 2011
		Tomato leaf curl purple vein virus	ToLCPVV	ssDNA	No	–	Brazil	Macedo et al., 2018
		Tomato leaf deformation virus	ToLDeV	ssDNA	No	–	Peru	Márquez-Martín et al., 2011
		Tomato mottle wrinkle virus	ToMoWV	ssDNA	No	–	Argentina	Vaghi Medina et al., 2015
		Tomato rugose yellow leaf curl virus	ToRYLCV	ssDNA	No	–	Uruguay	Márquez-Martín et al., 2012
		Tomato twisted leaf virus	ToTLV	ssDNA	No	–	Venezuela	Romay et al., 2019
		Tomato vein clearing leaf deformation virus	ToVCLDeV	ssDNA	No	–	Argentina	Vaghi Medina et al., 2020
		Tomato wrinkled mosaic virus	ToWMV	ssDNA	No	–	Venezuela	Romay et al., 2018
		Tomato yellow margin leaf curl virus	TYMLCV	ssDNA	No	–	Venezuela	Nava et al., 2013
	Unassigned	“Tomato associated geminivirus 1”	TaGV1	ssDNA	Yes	Total DNA-RCA/HiSeq 2500	Brazil	Fontenele et al., 2017
*Genomoviridae*	*Gemycirculavirus*	“Plant-associated genomovirus 2”	–	ssDNA	Yes	Total DNA-RCA/HiSeq 2500	Brazil	de Nazaré Almeida dos Reis et al., 2020
*Iflaviridae*	*Iflavirus*	Tomato matilda virus	TMaV	(+)ssRNA	Yes	Total RNA/GA IIx	Australia	Saqib et al., 2015
*Kitaviridae*	*Blunervirus*	Tomato fruit blotch virus	ToFBV	( + )ssRNA	Yes	Total RNA-RD/unspecified	Italy, Australia	Ciuffo et al., 2020
*Phenuiviridae*	*Tenuivirus*	Melon chlorotic spot virus	MeCSV	(–)ssRNA	Yes	Small RNA/HiSeq	France	Lecoq et al., 2019
*Potyviridae*	*Potyvirus*	Tomato necrotic stunt virus	ToNStV^3^	(+)ssRNA	Yes	Small RNA/GA IIx	Mexico	Li et al., 2012
*Rhabdoviridae*	*Cytorhabdovirus*	Tomato yellow mottle-associated virus	TYMaV	(–)ssRNA	Yes	Small RNA/HiSeq 2500	China	Xu et al., 2017
*Tollecusatellitidae*	*Betasatellite*	Tomato leaf curl Hajipur betasatellite	ToLCHLB	ssDNA	No	–	India	Kumar et al., 2013
		Tomato leaf curl Togo betasatellite	ToLCTGB	ssDNA	No	–	Togo	Kon and Gilbertson, 2012
*Tospoviridae*	*Orthotospovirus*	Pepper necrotic spot virus	PNSV	(–)ssRNA	No	–	Peru	Torres et al., 2012
		Tomato necrotic ringspot virus	TNRSV	(-)ssRNA	No	–	Thailand	Seepiban et al., 2011
		Tomato necrotic spot virus^4^	TNSV	(–)ssRNA	No	–	China	Yin et al., 2014
*Tymoviridae*	*Tymovirus*	Tomato blistering mosaic tymovirus	ToBMV	(+)ssRNA	No	–	Brazil	De Oliveira et al., 2013
*Virgaviridae*	*Tobamovirus*	Tomato brown rugose fruit virus	ToBRFV	(+)ssRNA	No	–	Jordan	Salem et al., 2016
		Tomato mottle mosaic virus	ToMMV	(+)ssRNA	Yes	Small RNA/HiSeq 2000	Mexico	Li et al., 2013

*Viruses are grouped according to virus family and genus, with its genome type indicated. The sample and library preparation approach and sequencing platform used are indicated for viruses discovered using HTS. Lastly, the country where the virus was first discovered and the corresponding publication and year are also indicated. ^1^ssDNA: circular, positive sense, single-stranded DNA (Group I), (+)ssRNA: linear, positive sense, single-stranded RNA (Group IV), (–)ssRNA: linear, negative sense, single-stranded RNA (Group V); ^2^total DNA-RCA: total DNA with rolling circle amplification, total RNA-RD: total RNA with rRNA depletion; ^3^Taken from recent literature, ToNSV and TNSV were previously also used in literature; however, they are also used for other viruses. ^4^The same name is used also for another virus from the genus Ilarvirus (family Bromoviridae).*

### Discovery and Generic Detection of Viruses in Tomato Using HTS

Fourteen new tomato viruses or virus satellites were detected using HTS in recent years (Table 1). As an example, HTS was used to characterize the diversity of viruses and viroids in tomato-growing areas of Mexico, where a novel tobamovirus, tomato mild mottle virus (ToMMV), and a novel potyvirus, tomato necrotic stunt virus (ToNStV), were discovered (Li et al., 2012, 2013). In the extensive study of the diversity of tomato viruses in China, a novel cytorhabdovirus, named tomato yellow mottle-associated virus (TYMaV), was discovered (Xu et al., 2017). In France, a novel ilarvirus was found in tomatoes and *Solanum nigrum*, named Solanum nigrum ilarvirus 1 (SnIV1) (Ma et al., 2020). Three begomoviruses, a gemycircularvirus, and a new alpha satellite were discovered by HTS in tomatoes in Brazil (de Nazaré Almeida dos Reis et al., 2020). A novel begomovirus named tomato-associated geminivirus 1 (TaGV1) was discovered using HTS in Brazil and was suggested as a member of a putative new genus closely related to *Capulavirus* (Fontenele et al., 2017). In Italy and Australia, a new blunervirus (*Kitaviridae*), named tomato fruit blotch virus (ToFBV), was discovered by HTS (Ciuffo et al., 2020). Tomato matilda virus (TMaV) (*Iflaviridae*), first detected in Australia using HTS (Saqib et al., 2015), was also detected in tomatoes grown in Italy, based on sequences deposited in GenBank (accession number MK517476) (Table 1 and Figure 1). The infectivity of ToFBV and TMaV in tomato, other biological characteristics, and their impact on tomato yield are not yet known.

Aside from discovery of new viruses, HTS was also used to detect known viruses for the first time in tomato. In China, six known viruses, namely, potato virus A, tobacco vein banding mosaic virus (*Potyviridae*), potato virus H, potato virus S, potato virus M (*Betaflexiviridae*), and turnip yellows virus (*Luteoviridae*) were reported for the first time in tomato (Xu et al., 2017). In Germany, Physostegia chlorotic mottle virus (PhCMoV) was detected for the first time in tomato using HTS (Gaafar et al., 2018), and in the United States, cherry rasp leaf virus was detected (Bratsch et al., 2020) in tomato. In a survey of tomato and pepper viruses in Vietnam, pepper chlorotic spot orthotospovirus (*Tospoviridae*) and Lindernia anagallis yellow vein virus (*Geminiviridae*) were associated with tomato for the first time (Choi et al., 2020). A known virus, discovered in henbane in 1932 in England, called henbane mosaic virus (HMV), but later on rarely detected, was found using HTS for the first time in tomatoes from Slovenia (Pecman et al., 2018). In the same study, HTS was also used to reconstruct complete genomic sequences of the isolate from tomato and historic isolates from virus collections, since only partial genomic sequence of the virus was available before.

### Post-discovery Characterization of New Tomato Viruses

**FIGURE 2:**
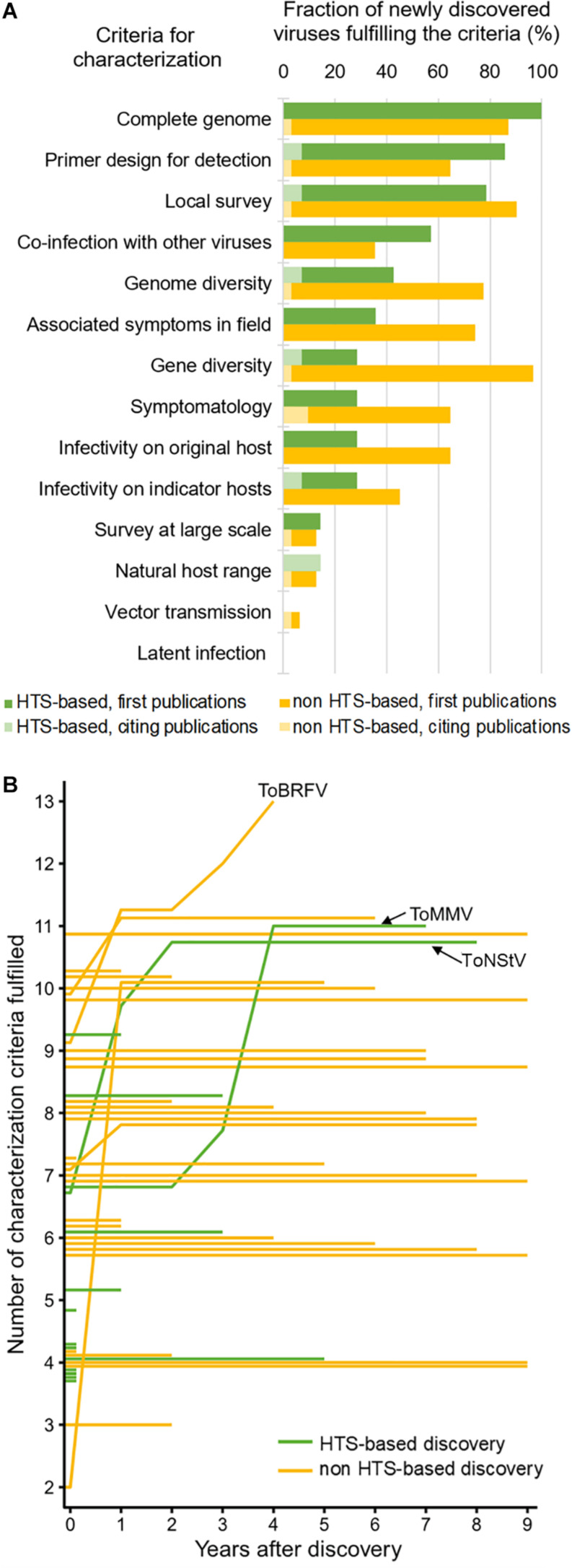
Post-discovery characterization of new tomato-infecting viruses according to the literature review. Percentages of newly discovered virus species fulfilling each of the 14 characterization criteria are shown for viruses discovered by HTS or other methods **(A)**. Fulfillment of characterization criteria plotted through the years elapsed since the first report is shown as a line plot **(B)**, wherein each line represents a single virus and the evolution of its characterization; the lines corresponding to the emerging tomato brown rugose fruit virus (ToBRFV), and the first two tomato viruses discovered through HTS, tomato mottle mosaic virus (ToMMV) and tomato necrotic stunt virus (ToNStV), are labeled.

Even though HTS advanced our ability for generic detection and discovery of novel viruses, it can only provide us with genomic information of the virus. For a meaningful biological characterization, classical virology tools are still very much needed, which include plant bioassays (i.e., infectivity and transmission assays), electron microscopy, and targeted diagnostics. Virus characterization is a time- and resource-intensive task that often requires efforts from different institutions, sometimes at an international scale. A framework for biological characterization of viruses discovered by HTS was proposed to facilitate this part of the research pipeline (Massart et al., 2017). We used this framework to review how the 45 new viruses discovered in tomato, both through HTS and through other methods, were characterized. The same approach was recently employed for fruit tree infecting viruses (Hou et al., 2020), where fulfillment of 14 characterization categories were reviewed. We analyzed 53 publications on novel tomato viruses (41 reporting viruses for the first time and 12 follow-up studies) from the 2011–2020 period contributing to characterization of newly discovered viruses associated with tomato, reviewing the fulfillment of 14 characterization categories (Figure 2A; for details, see Supplementary Table 1).

Our review showed that, e.g., for majority (>60%) of the HTS-based new virus discoveries, whole genome sequence was determined, primers for detection of the virus with PCR were designed, local survey of prevalence was done, and co-infection with other viruses was checked (Figure 2A). Comparing HTS-based and non-HTS-based virus discoveries, there were fewer infectivity studies done on the original host and other natural or indicator hosts for HTS-based discoveries compared to that of non-HTS discoveries. Moreover, vector transmission was studied in very few cases and possibility of latent infections has not been studied for any of the newly discovered viruses. Practically, completeness of virus characterization greatly depended on the phytosanitary priority level of a virus. For tomato brown rugose fruit virus (ToBRFV), which is an emerging virus with significant economic importance, 13 of 14 characterization criteria were fulfilled in less than 4 years after the discovery, included in five publications after its first report. On the other hand, ToNStV and ToMMV, which were the first tomato-infecting viruses discovered using small RNA sequencing, were more extensively characterized after 7–8 years. Finally, many viruses were not followed up at all after the initial discovery and first publication (Figure 2B).

### Looking Outside Tomato: Vectors, Weeds, Water, and Epidemiological Implications of HTS

Spatially, crop lands are always in conjunction with the wild or urban ecosystems bound by an agroecological interface, which is characterized by active biological interactions and exchange of materials, including pests and pathogens (Alexander et al., 2014). Nevertheless, it was just in the recent decade that plant virologists started to explore more intensively the diversity of viruses in wild ecosystems. Plant virus ecology and epidemiology include studies on viruses infecting weeds and other wild plants (Malmstrom and Alexander, 2016; Shates et al., 2019) as well as water as potential channel for plant virus spread (Mehle and Ravnikar, 2012). In this context, HTS is a well-suited tool, which allows generic investigation of viromes in a diverse set of sample matrices, from an array of different plant species to environmental samples, such as water and soil.

Weeds and other wild plants could act as reservoirs from which plant viruses can spill to crops, or vice versa (Malmstrom and Alexander, 2016). The association of tomato viruses with weeds also implies increased chances of survival for the viruses because weeds have high reproductive rates and are environmentally persistent (Cooper and Jones, 2006). These findings imply importance of weeds in plant-virus pathosystems and the importance of their inclusion in epidemiological studies. A recent review of viruses infecting plants from the *Solanaceae* family highlights the importance of studying the virome of both wild and cultivated plants to fully understand the plant virus impact on the agroecological scale (Hančinský et al., 2020). For tomato-infecting viruses, several studies were done to elucidate the role of wild plants as virus reservoirs, more specifically referred to in Section *“*Emerging and endemic viruses and viroids causing significant economic damages in tomato production in the past decade*.”*

The first extensive tomato virome HTS-based study was done in major tomato-growing areas in China (Xu et al., 2017), resulting in a lot of new knowledge about occurrence of tomato viruses in the region and revealed several previously unknown associations of known plant viruses with tomato. It also suggested that some viruses originally associated with insect hosts (Wuhan insect virus 4, 5, and 6) (Li et al., 2015) might in fact be plant viruses, since they were found in tomato and they cluster with other plant viruses in phylogenetic analysis (Xu et al., 2017). This study did not look into virome of wild plants growing nearby. On the other hand, a recent large-scale virome study of tomato in France also included its wild relative, *S. nigrum* or European black nightshade (Ma et al., 2020). The study suggested the likely exchange of some viruses between tomato and *S. nigrum*; a newly discovered SnIV1 was found in both species, as well as potato virus Y (PVY), for which there was a strong evidence for a likely spillover from tomato to *S. nigrum*. On the other hand, a possible biological and ecological barrier was proposed for a spread of broad bean wilt virus (known to be able to infect both hosts) from *S. nigrum* to tomato, since high incidence and diversity of this virus was observed in *S. nigrum*; however, the virus was not detected in tomato in this study. Moreover, discovery of Euphorbia caput-medusae latent virus by HTS in South Africa in wild plant *Euphorbia caput-medusae*, which was shown to be infective in tomato and *Nicotiana benthamiana*, led to the establishment of a new genus, *Capulavirus*, in the family *Geminiviridae* (Bernardo et al., 2013).

Even prior to the application of HTS-based methods for detection of plant viruses, several studies detected plant viruses outside of the host, in environmental samples, such as water (Mehle and Ravnikar, 2012) and in soil (Fillhart et al., 1998). Several plant viruses are stable in the environment and can survive long periods of time outside the host (Mehle et al., 2018). Water can serve as virus transmission channel that have the potential to create new infection foci and to spread viruses in a wide range of host species and at long distances (Jones, 2018). HTS-based virome studies of different environmental waters all over the world reported the presence of plant virus nucleic acids in various types of samples. With the use of HTS, viruses in wastewater, irrigation water, and potable water can be monitored in extended areas over a regular period of time, which can provide a useful info for predicting or following disease outbreaks (Mehle et al., 2018). A recent virome study focusing on plant viruses in wastewater in Slovenia gave insights into diverse economically important plant viruses that might be circulating in the area. Wastewater treatment plant influents and effluents contained nucleic acids of at least 47 viruses, both previously known and also unknown to be present in the region. Tobamoviruses were highly represented in the samples, and for three of them, all of which can infect tomato [pepper mild mottle virus, tobacco mild green mosaic virus and tomato mosaic virus (ToMV)], the infectivity was confirmed (Bačnik et al., 2020). Alongside other detected plant viruses, nucleic acids of ToBRFV, which is currently one of the major threats in tomato production worldwide, were also detected in this study; however, ToBRFV was still not detected in the plants in the area. Moreover, pollinators, specifically honeybees (*Apis mellifera*), were studied as possible effective ecological “samplers” of plant viruses. HTS was employed in a recent study of honeybee-assisted surveillance in Australia to investigate the presence of plant viruses, and cucumber green mottle mosaic virus was detected on bees in several states of Australia before its subsequent detection in diseased plant material (Roberts et al., 2018).

High-throughput sequencing can also be used to rapidly obtain complete genome sequences of many viral isolates of the same species and thus follow the molecular epidemiology of viruses in real time. As such, HTS was used in a recent comprehensive study of whole genome sequences of ToBRFV from the Netherlands, which revealed the existence of three virus phylogenetic clusters in the country, which are hypothesized to represent three different introduction sources of the virus (van de Vossenberg et al., 2020).

## Emerging and Endemic Viruses and Viroids Causing Significant Economic Damages in Tomato Production in the Past Decade

**FIGURE 3:**
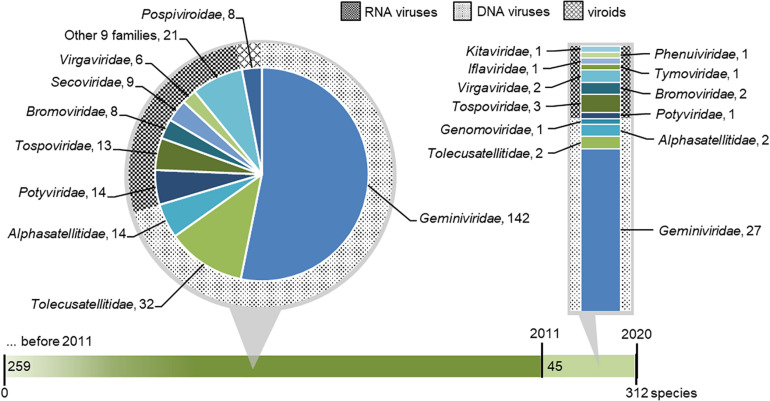
Taxonomic distribution of virus and viroid species that were reported to infect or were associated with tomato. The green horizontal bar represents tomato viruses discovered before 2011 and within the 2011–2020 period. Known species reported before 2011 (including all known viruses that were first associated in tomato within the 2011–2020 period) are shown in the dark green portion on the left and extended in pie chart callout, where their distribution across viral families is shown. Species newly discovered in 2011–2020 are shown in the light green portion of the bar and extended in bar chart callout, where their distribution across viral families is shown. In both callouts, families of DNA viruses, RNA viruses, and viroids are designated with differently hatched backgrounds.

We collected a list of viruses and viroids with tomato indicated as host (Supplementary Table 2). A total of 312 species of tomato viruses (including satellite viruses) and viroids were verified. They are classified across 39 genera and 22 families (Figure 3). Among the species in the list, 220 species of tomato viruses (including satellite viruses) have DNA genomes and are classified in three families. Due to the high species count within a genus, the majority of viral species infecting tomato belongs to the *Begomovirus* genus (DNA viruses) and associated satellites. On the other hand, a greater richness of tomato viruses is found for RNA viruses, for which 84 known species are classified in 18 families. Finally, eight viroids, from a single family, are known to infect tomato (Figure 3).

Virus can be considered as emerging if it recently has changed or appeared to occupy and spread within a new niche (Rojas and Gilbertson, 2008). In general, emergence is connected to ecological change or intensive agricultural practices (Elena et al., 2014). Complex virus epidemiology, especially insect transmission (by, e.g., whiteflies, aphids, and thrips), and constant evolution of virus populations are key factors associated with emergence and outbreaks of viruses. Emerging pathogens and pests in crop plants are often accompanied by significant yield or economic losses (Savary et al., 2019). In addition, changes in global climate conditions, e.g., increasing temperatures, are predicted to generally exacerbate spread of plant virus diseases in many regions and can cause changes in the severity of the disease symptoms (Canto et al., 2009; Jones, 2016, 2021; Trebicki, 2020). As an example, tomato yellow leaf curl virus (TYLCV) invasion risk analysis under different climate scenarios predicted emergence of the virus and its primary vector, *B. tabaci*, in tomatoes cultivated in open fields worldwide (Ramos et al., 2019). Another study showed that, indeed, heat stress accompanied by the infection of TYLCV resulted in lower stress response efficiency in tomatoes and higher aggregation of TYLCV proteins and DNA (Anfoka et al., 2016). Higher day–night temperature regimes were also shown to favor virion accumulation of tomato spotted wilt orthotospovirus (TSWV) in solanaceous weeds (Llamas-Llamas et al., 1998), and pepper plants that are heterozygous for the *Tsw* resistance gene showed susceptibility at higher temperatures (Moury et al., 1998). Pepper plants with tobamovirus resistance genes were also shown to be susceptible to ToBRFV, when grown in infected soil at 32°C (Luria et al., 2017).

Over the past decades, significant damages to tomato production were caused by viruses described as emerging or re-emerging, such as a few criniviruses, pepino mosaic virus (PepMV), tomato torrado virus (ToTV), tomato leaf curl New Delhi virus (ToLCNDV), and ToBRFV (Hanssen et al., 2010b; Navas-Castillo et al., 2011; Gómez et al., 2012; Moodley et al., 2019, 2020; Oladokun et al., 2019). On the other hand, significant yield losses in tomato could also be associated with widespread, endemic viruses, such as PVY or cucumber mosaic virus (CMV) (Quenouille et al., 2013; Tepfer and García-Arenal, 2019). In the following sections, we review the available information about an array of important emergent or endemic viruses and viroids in tomato, as well as some recently discovered viruses with yet unclear role in tomato health.

### Begomoviruses and Their Satellite Viruses

*Begomovirus* (*Geminiviridae*) is the biggest and one of the most studied genera of plant viruses, comprising 162 known species infecting tomato. Seven viral species belonging to other genera from the family *Geminiviridae* are also known to infect tomato (Figure 3). Virions of viruses belonging to *Geminiviridae* are typically twinned (“geminate”). Begomoviruses have monopartite or bipartite genomes (Zerbini et al., 2017). The most important way of the transmission of begomoviruses is by the whiteflies *Bemisia tabaci*, a cryptic species complex, which can infest many crops and is now globally widespread (Stansly et al., 2010; Fiallo-Olivé et al., 2020).

Among the tomato-infecting begomoviruses, ToLCNDV and TYLCV are the most widespread and economically important (Zaidi et al., 2017; Prasad et al., 2020). ToLCNDV is a re-emerging bipartite begomovirus known to infect around 47 different plant species, predominantly crops and weeds from *Solanaceae* and *Cucurbitaceae* (Moriones et al., 2017) families. ToLCNDV, distinct species with two strains (ToLCNDV and ToLCNDV-ES), was first described in India (Padidam et al., 1995) and it is mainly present in Asia where many host plants and various isolates were described (Zaidi et al., 2017). In the Mediterranean basin, the ToLCNDV-ES recombinant strain was identified in tomatoes showing stunting, yellowing, and/or curling in apical leaves (Fortes et al., 2016). ToLCNDV was then reported to spread in Africa and in European countries (e.g., Spain, Italy, Greece, Portugal) (EPPO GD, 2020). Furthermore, the monopartite TYLCV has several widespread strains that are altogether considered as most devastating for tomato production in the tropical and subtropical tomato-growing regions (Mabvakure et al., 2016). Severe yield losses of up to 100% were frequently recorded in tomato yellow leaf curl virus-infected susceptible cultivars showing typical symptoms, such as stunting, upward curling of leaves, chlorosis, and reduction in leaf size (Prasad et al., 2020). For instance, losses due to TYLCV outbreaks in the Dominican Republic were estimated at over 10 million US dollars (Gilbertson et al., 2007), and in China, the virus was reported to be widely spreading and causing unprecedented losses (Pan et al., 2012). Several resistance genes (e.g., *Ty-1, Ty-2, Ty-3*) identified from wild tomato relatives are used to breed for tolerant varieties against ToLCNDV, TYLCV, and other begomoviruses and multi-gene resistance is currently considered to increase the level of resistance and prevent its breakdown (Zaidi et al., 2017; Prasad et al., 2020).

Circular ssDNA satellite viruses (i.e., alphasatellites, betasatellites, and deltasatellites) are often associated with begomoviruses (Rao and Kalantidis, 2015). They can influence helper virus (i.e., begomovirus) multiplication and disease severity (Gnanasekaran and Chakraborty, 2018). Their replication, cell-to-cell movement and vector transmission are dependent on helper virus, except for independently replicating alphasatellites (Gnanasekaran and Chakraborty, 2018). Several betasatellites were connected with enhancement of symptom severity of TYLCV, e.g., cotton leaf curl Gezira betasatellite, tobacco leaf curl Japan betasatellite, honey-suckle yellow vein mosaic betasatellite (Ito et al., 2009), and tomato leaf curl Philippines betasatellite (Sharma et al., 2011). Co-inoculation experiments of several betasatellites with ToLCNDV resulted in enhancement of symptom severity (Jyothsna et al., 2013), while, in one experiment, co-inoculation of an alphasatellite, betasetellite, and TYLCV resulted in reduction of the accumulation of betasatellite and symptoms induced by TYLCV (Idris et al., 2011) in *N. benthamiana*.

In the last 10 years, several novel begomovirus species were described in different parts of the world (Table 1 and Figure 1). From the Afrotropic ecoregion, tomato leaf curl Kunene virus (Lett et al., 2020), tomato leaf curl Mahé virus (Scussel et al., 2018), and tomato leaf curl Burkina Faso virus (Ouattara et al., 2017) were recently discovered. Numerous begomoviruses were discovered in Neotropic countries, which include four novel species: tomato leaf deformation virus (ToLDeV; Márquez-Martín et al., 2011), tomato apical leaf curl virus (Vaghi Medina et al., 2018), tomato twisted leaf virus (Romay et al., 2019), and pepper leafroll virus (PepLRV; Melgarejo et al., 2013; Martínez-Ayala et al., 2014). Initial surveys suggest that ToLDeV and PepLRV are the predominant pathogens causing the tomato leaf curl disease complex in the Ecuadorian and Peruvian regions (Melgarejo et al., 2013). In Brazil, tomato severe rugose fruit virus (ToSRV) and tomato mottle leaf curl virus (ToMoLCV) were found to be prevalent in tomato crops (Souza et al., 2020).

Reports were made on the potential of wild plants to act as hosts for important tomato-infecting begomoviruses (Prajapat et al., 2014). In Cyprus, a large-scale survey of more than a hundred weed species in major tomato-growing areas revealed the presence of TYLCV in 49 species (in 15 families), suggesting inclusion of these possible reservoir hosts in control measures (Papayiannis et al., 2011). A recent study focused on ToLCNDV found the virus in 5 out of 24 investigated wild plant species in Spain. In that study, HTS was used to characterize the genomes of 80 isolates from different plant species and analyze within-plant virus population diversities for some of them, hinting at some differences between the isolates from wild and cultivated (cucurbit) plants. Tomato chlorotic mottle virus (TCMV) was also found in several weed species in Brazil (Ambrozevicius et al., 2002). In Spain, *S. nigrum* was shown to harbor four known begomoviruses associated with tomato yellow leaf curl disease. Through infectivity assays, tomato severe rugose virus was shown to be infective and harbored in common weeds, such as *Nicandra physaloides*, *Datura stramonium*, and *Sida* sp., which are found surrounding the tomato-growing areas in Brazil (Barreto et al., 2013; Gorayeb et al., 2020).

### Tobamoviruses

Tobamoviruses (*Virgaviridae*) are among the most environmentally stable viruses that remain viable for a long time outside of their host plant in various environmental media (e.g., water, soil) (Fillhart et al., 1998; Mehle and Ravnikar, 2012). A positive-sense single-stranded RNA [(+)ssRNA] genome of tobamoviruses is around 6.5 kb long and coated in rigid rod virion (Adams et al., 2009b). They are efficiently transmitted mechanically (e.g., by wounding and contact) and can be spread also passively through water and soil, and vertically through seeds (Dombrovsky and Smith, 2017; Mehle et al., 2018). Six tobamoviruses are known to infect tomato, and two of them are known to be globally widespread for many years, namely, tobacco mosaic virus (TMV) and ToMV. Since the discovery and deployment of three resistance genes, *Tm-1*, *Tm-2*, and *Tm-2*^2^, the economic damages caused by these two viruses reduced dramatically (Pfitzner, 2006).

The most recent virus outbreaks with significant losses in tomato production worldwide were connected to the emergence of two new tobamovirus species, ToMMV (Li et al., 2013, 2017; Sui et al., 2016) and ToBRFV (Salem et al., 2016; Luria et al., 2017). Many research efforts are directed in studies of these viruses, particularly ToBRFV, and significant efforts are directed to limit their spread with appropriate quarantine and phytosanitary measurements (EPPO, 2020a, b).

ToBRFV was first detected in field-cultivated tomatoes showing typical mosaic symptoms as well as narrowing of leaves (Luria et al., 2017) and strong brown rugose spots on fruits (Salem et al., 2016). ToBRFV was found in tomatoes in Jordan in 2015 (Salem et al., 2016) and in 2014 in tomatoes from Israel, where ToBRFV was also shown to break the *Tm-2*^2^ resistance in some tomato cultivars (Luria et al., 2017). ToBRFV likely emerged as a recombinant between TMV and ToMMV (Salem et al., 2016). It can infect up to 100% of plants at a location and amounts to around 70% tomato yield losses due to symptoms expressed in the fruits (EPPO, 2020a). After its first detection in Israel and in Jordan, it has rapidly spread into many other countries (Figure 1). It was recently reported in the United States (Ling et al., 2019), Mexico (Camacho-Beltrán et al., 2019), China (Yan et al., 2019), Palestine (Alkowni et al., 2019), Turkey (Fidan et al., 2019), Germany (Menzel et al., 2019), Netherlands (van de Vossenberg et al., 2020), France (Verdin et al., 2020), Greece (Beris et al., 2020), Italy (Panno et al., 2019), the United Kingdom (Skelton et al., 2019), Egypt (Amer and Mahmoud, 2020), and Spain (Alfaro-Fernández et al., 2021). ToBRFV can be easily transmitted through mechanical contact or can be transmitted by bumblebees (Levitzky et al., 2019), similar to what was reported for TMV (Okada et al., 2000). ToBRFV can overcome known resistance genes against tobamoviruses and there are currently no commercially available ToBRFV-resistant tomato varieties; thus, it remains an imminent threat to tomato production worldwide (EPPO, 2020a). Currently, the only possible control measures are the ones directed at eradication and containment of the infections, such as restriction of access to the production site, disposal of infested plant material, and sanitation measures (EPPO, 2020a). Moreover, a recent research reported that co-infection of ToBRFV with mild strain PepMV resulted in enhanced PepMV accumulation and symptoms characteristic for an aggressive PepMV strain (Klap et al., 2020), bringing additional concerns about the impact of ToBRFV on tomato production.

ToMMV was first isolated from infected greenhouse-grown tomatoes showing mosaic and leaf distortion in Mexico in 2013 (Li et al., 2013). Later on, ToMMV was reported to occur across three ecoregions, specifically in the United States (Padmanabhan et al., 2015a), Israel (Turina et al., 2016a), Spain (Ambrós et al., 2017), China (Che et al., 2018), Brazil (Nagai et al., 2019), Iran, and Czech Republic (EPPO GD, 2020). Interception of ToMMV introduction in Australia through infected pepper seeds was made in biosecurity checks (Lovelock et al., 2020), highlighting the importance of preventive measures in preventing virus spread. ToMMV was shown to successfully infect a small portion of ToMV-resistant plants (Sui et al., 2016) but did not successfully infect some cultivars with *Tm-2*^2^ gene, (Nagai et al., 2019). Further assessment is needed to ascertain yield loss and economic impact of ToMMV.

Through mechanical inoculation, ToBFRV was shown to be able to infect various hosts, aside from crops from genus *Solanum*, also *Chenopodium* spp., *Petunia hybrida*, and wild relative of tomato, *S. nigrum* (Luria et al., 2017; Alkowni et al., 2019; Yan et al., 2019). ToMMV was experimentally shown to infect various species from family Solanaceae (Ambrós et al., 2017), causing systemic necrosis and death in *Datura stramonium* (Sui et al., 2016). No further studies were conducted yet on the possibility of these alternative hosts to act as natural reservoir of these viruses.

### Orthotospoviruses

The genus *Orthotospovirus* (*Tospoviridae*) is currently composed of 26 species and is the only plant-infecting genus in the order *Bunyavirales* (Oliver and Whitfield, 2016; ICTV, 2020). Orthotospoviruses have a single-stranded negative-sense RNA [(−)ssRNA] genome, divided into three segments, and are capable of replication both in the plant host and in thrips (Riley et al., 2011). Economically, the most important orthotospovirus infecting tomato is the re-emerging TSWV. In the case of TSWV outbreaks, up to 95% losses in total market value can be recorded due to tomato fruits showing typical necrotic spots and concentric rings (Sherwood et al., 2003; Sevik and Arli-Sokmen, 2012).

TSWV has one of the widest host ranges of all plant viruses with more than 1000 known host species from 85 families (Parrella et al., 2003). In large-scale surveys of weeds in United States, several were proven to harbor TSWV, and, among them, *Sonchus asper*, *Taraxacum officinale*, *Ranunculus sardous*, *Aster* sp., *Senecio vulgaris*, and *S. nigrum* were shown to have the highest potential to be an inoculum source of TSWV (Chatzivassiliou et al., 2001; Groves et al., 2002). Four weed species surrounding tomato-growing areas in Kenya were also shown to be hosts of TSWV (Macharia et al., 2016). TSWV was found in all known ecozones of the world, affecting numerous crops (Butković et al., 2021). Orthotospoviruses are transmitted in a persistent propagative and circulative manner by thrips, the most important of which is western flower thrips (*Frankliniella occidentalis*). This insect vector remains a global problem due to inefficient insecticidal control (Riley et al., 2011), thus contributing to continuous emergence of orthotospoviruses worldwide (Oliver and Whitfield, 2016). Several resistance genes have been identified and employed to confer resistance against TSWV in tomato, *Sw-5b* offering the most durable and broad resistance to different orthotospovirus species (Turina et al., 2016b).

Tomato spotted wilt orthotospovirus resistance-breaking (RB) strains were first discovered in the early 2000s, and since then, they are occurring sporadically in many regions causing significant damages. These strains overcome TSWV resistance regulated by hypersensitivity genes: *Tsw* (in pepper) and *Sw-5* (in tomato). This further worsened the economic losses caused by TSWV in tomato and pepper, which were previously estimated at more than 1 billion US dollars annually (Prins and Goldbach, 1998). Currently, there is no new resistance gene discovered against TSWV (Turina et al., 2016b).

Among other orthotospoviruses, important viruses reported in tomato in the past decade are tomato chlorotic spot virus (TCSV), groundnut ringspot virus (GRSV), capsicum chlorosis virus (CaCV), and tomato yellow ring virus (TYRV) (Table 1 and Figure 1). TCSV was reported to be actively spreading in the Nearctic countries such as United States (Sui et al., 2017; Poudel et al., 2019), Puerto Rico (Webster et al., 2013) and GRSV was reported in tomatoes and other vegetables in the United States (Webster et al., 2010, 2014). CaCV is expanding its distribution in Indo-Malay and Australasian ecoregions as it was detected in Taiwan (Huang et al., 2010), China (Yin et al., 2015), and Australia (Sharman et al., 2020). TYRV was reported in Kenya (Birithia et al., 2012) and Poland (Zarzyñska-Nowak et al., 2016). Other novel orthotospoviruses recently identified are tomato necrotic ring virus (TNRV) from Thailand (Hassani-Mehraban et al., 2011), pepper necrotic spot virus (PNSV) from Peru (Torres et al., 2012), and tomato necrotic spot virus (TNSV; as a word of caution, an ilarvirus was named identically, but has another acronym: ToNSV) from China (Yin et al., 2014). These viruses were not yet associated with significant or large-scale outbreaks.

### Potyviruses

*Potyvirus* (*Potyviridae*) is one of the largest genera of plant-infecting viruses, with 183 recognized species (ICTV, 2020), 15 of which are known to infect tomato (Figure 3). Potyviruses are characterized by a (+)ssRNA genome (∼9.7 kb), which is coated in a flexible filamentous virion. PVY is an economically important potyvirus infecting solanaceous crops. It is efficiently transmitted by *Myzus persicae* and other aphid species (Revers and García, 2015; Gadhave et al., 2020). PVY is widespread in several tomato-growing regions worldwide, based on recent large-scale studies (Soler et al., 2010; Al-Kuwaiti et al., 2016; Xu et al., 2017; Nikolić et al., 2018; Ignatov et al., 2020; Ma et al., 2020). Recently, PVY strain C was detected in tomatoes showing leaf necrosis in Kenya (Chikh-Ali et al., 2015). Symptoms of PVY infection in tomato ranges from faint mottling to necrosis but varies depending on plant age, environmental conditions, PVY strain, and co-infecting viruses (Sastry et al., 2019). Aside from tomato, PVY was shown to infect weed species from *Asteraceae, Chenopodiaceae, Geraniaceae*, and *Lamiaceae* families (Kaliciak and Syller, 2009).

In recent years, three other potyviruses infecting tomato were reported, namely, chili veinal mottle virus (ChiVMV), pepper mottle virus (PepMoV), and pepper veinal mottle virus (PVMV). PepMoV infection in tomatoes in Hawaii resulted in unmarketable fruits with mottling symptoms (Melzer et al., 2012) and, recently, the virus was detected in India (Sharma et al., 2019). ChiVMV was detected in several provinces in southwest China, with recorded incidence of up to 90% in some areas (Zhao et al., 2014; Zhu et al., 2017). PVMV was reported to cause considerable epidemics in tomato-growing areas in Mali in 2010 and is now under consideration in tomato disease resistance breeding programs (Tsai et al., 2010). HMV was recently reported to naturally infect field tomatoes in Slovenia (Pecman et al., 2018).

### Cucumoviruses and Other Members of Family *Bromoviridae*

Cucumber mosaic virus (genus *Cucumovirus*, family *Bromoviridae*) is an important tomato-infecting virus. Cucumoviruses have a spherical virion encapsidating a tripartite (+)ssRNA genome. Some CMV strains encapsidate subviral RNAs termed satellite RNAs (satRNAs) (Masuta, 2014). In nature, CMV and other cucumoviruses are non-persistently transmitted by aphid species, the most important of which are *Myzus persicae* and *Aphis gossypii.* They were also shown to be transmitted through seeds of some species, but not tomato (Palukaitis and García-Arenal, 2003). CMV has a very broad host range, able to infect more than 1200 plant species across 100 botanical families (Mochizuki and Ohki, 2012). Several weed species surrounding tomato production areas were shown to harbor CMV and could serve as reservoir or alternative host of the virus. Such weeds include, e.g., *Convolvulus arvensis*, *Malva sylvestris*, and *Sonchus tenerrimus*, reported from Spain (Lavina et al., 1996), and other nine diverse weeds from Pakistan (Akhtar et al., 2019). Due to its very broad host range, transmissibility through seeds of some species, and ubiquitous presence of its aphid vectors (Jacquemond, 2012), CMV became a globally distributed species that still causes significant damages in many crops, especially in tomato.

Outbreaks of CMV infection in tomato-growing areas were reported, and some of these were associated with satellite RNAs (satRNAs) that induce systemic necrotic symptoms, referred to as lethal necrosis (Garcìa-Arenal and Palukaitis, 1999; Xu et al., 2003). This disease was reported in Italy between 1988 and 1993 (Cillo et al., 1994; Grieco et al., 1997) and in Croatia in 1993 (Škoric et al., 1996). Recently, CMV-satRNA infection was reported in legume crops in Greece (Chatzivassiliou et al., 2016), and was shown to cause lethal necrosis in tomato through mechanical inoculation (Giakountis et al., 2018). Also, during a 4-year survey in Serbia (2012–2015), CMV satRNAs were identified from collected tomato samples showing systemic necrosis accompanied by fruit malformation (Stanković et al., 2021). Cultivated crops are important reservoir of CMV satRNAs (Gallitelli, 2000), and thus, strategic intercropping should be taken into consideration to prevent devastating CMV lethal necrosis outbreaks in tomato.

Tomato aspermy virus (TAV) is another cucumovirus that naturally infects tomato. TAV was recently reported in tomato samples from China; however, its economic impact was not recorded (Xu et al., 2017). TAV was reported as a major pathogen of ornamentals such as chrysanthemums that are grown in sub-tropical Asia (Verma et al., 2009; Maddahian et al., 2017).

Tomato necrotic spot virus (ToNSV) and the novel species named tomato necrotic streak virus (TomNSV) are tomato-infecting ilarviruses within *Bromoviridae* family. Detections of these viruses were reported in the United States (Batuman et al., 2009; Adkins et al., 2015; Badillo-Vargas et al., 2016; Bratsch et al., 2018, 2019). Both viruses caused necrotic symptoms in leaves, stem or fruits of tomatoes. In France, a novel ilarvirus, named SnIV1, was discovered in both tomato and its wild relative, *S. nigrum* (Ma et al., 2020). However, the transmission routes, possible reservoir or alternative hosts, and economic impact are yet to be determined for ToNSV, TomNSV, and SnIV1 (Badillo-Vargas et al., 2016; Bratsch et al., 2019; Ma et al., 2020). Moreover, a re-emerging ilarvirus named parietaria mottle virus (PMoV) was suggested to be a threat to tomatoes, which showed rings and bright necrotic mosaic on young leaves upon infection. It was reported in Europe, particularly in the Mediterranean countries such as Italy, France, Greece, and Spain (Aparicio et al., 2018). Outbreaks of PMoV were recently reported in Sardinia, Italy (Parrella et al., 2020) and research on possible sources of resistance against the virus already started (Parrella, 2020). Lastly, two other members of *Bromoviridae* known to infect tomato and a variety of other crops are alfalfa mosaic virus (*Alfamovirus*), causing necrotic yellows disease on tomato (Finetti Sialer et al., 1997; Nikolić et al., 2018) and pelargonium zonate spot virus causing mild mosaic, leaf malformation, and severe stunting of the plants (Finetti-Sialer and Gallitelli, 2003; Lapidot et al., 2010).

### Potexvirus: Pepino Mosaic Virus (PepMV)

PepMV belongs to genus *Potexvirus* (*Alphaflexiviridae*) and has an unsegmented (+)ssRNA genome coated in a flexuous rod virion. PepMV was first described in Peru, and since its first detection in tomato in Netherlands in 1999, it quickly spread in Europe (van der Vlugt et al., 2000; Hanssen and Thomma, 2010). It is efficiently transmitted mechanically (e.g., contaminated tools and whitefly feeding) (Hanssen and Thomma, 2010; Noël et al., 2014) with occasional transmission through seeds (Hanssen et al., 2010c), as well as through water (Mehle et al., 2014). PepMV was originally discovered to infect pepino (*Solanum muricatum*) (Jones et al., 1980), and it quickly became endemic in tomato since its first discovery in this plant in 1999 (van der Vlugt et al., 2000; van der Vlugt and Stijger, 2021). In tomato, fruit marbling symptoms are considered as the most important cause of significant economic losses around the world (Hanssen and Thomma, 2010; Hanssen et al., 2010b). PepMV is present in major tomato-growing areas of the Mediterranean (Gómez et al., 2012) and was recently reported in United States and Mexico (Ling and Zhang, 2011), South Africa (Carmichael et al., 2011), Spain and Morocco (Gómez-Aix et al., 2019), and Serbia (Stanković et al., 2020).

To date, there are five strains identified for PepMV, namely, (1) the Peruvian (LP) strain, originally found infecting pepino (*S. muricatum*) and wild *Solanum* spp.; (2) the European EU-tomato (EU) strain; (3) the American US1 strain; (4) the Chilean-2 (CH2) strain; and (5) the PES strain, described in wild tomato populations in Peru (Moreno-Perez et al., 2014). PepMV was reported to infect a wide range of crops and weed species (Gómez et al., 2012); its infectivity and symptom expression are also dependent on the virus strain and host species or cultivar (Kazinczi et al., 2005; Hanssen et al., 2009; Blystad et al., 2015).

Due to the extensive damages caused by PepMV infections, and since there are currently no commercially available PepMV-resistant tomato varieties available, efforts have been made to produce efficient cross-protection strategies against the virus, particularly for the strains circulating in Europe (Pechinger et al., 2019). Other than host resistance, cross-protection is one of the most effective methods of virus disease management, which entails “pre-immunization” of susceptible genotypes by inoculation of mild strain of a particular virus (Ziebell and Carr, 2010). Successful applications of PepMV mild strain cross-protection were reported in the laboratory or greenhouse in the past years (Hanssen et al., 2010a; Schenk et al., 2010; Vermunt and Kaarsemaker, 2017). Recently, successful protection of field tomatoes against PepMV were reported using two mild strains of the virus (Agüero et al., 2018).

### Criniviruses

Criniviruses from genus *Crinivirus* (*Closteroviridae*) are whitefly transmitted viruses with bipartite genome composed of two (+)ssRNA genome segments that are separately coated in filamentous virions (Kiss et al., 2013). Their infection in plants can be mistaken for nutritional disorders and phytotoxicity because of the obvious interveinal leaf yellowing and leaf brittleness, resulting in reduced overall yield (Tzanetakis et al., 2013). Two widespread tomato-infecting criniviruses are tomato infectious chlorosis virus (TICV) and tomato chlorosis virus (ToCV). TICV was first described in 1996 in the United States (Duffus et al., 1996), and recently reported to cause yield losses in Mexico (Méndez-Lozano et al., 2012), Greece (Orfanidou et al., 2014), and Serbia (Vučurović et al., 2019). ToCV was first described in 1998 (Wisler et al., 1998) and is now present globally causing yield losses due to fruit size reduction (Fiallo-Olivé and Navas-Castillo, 2019). ToCV was shown to infect 25 species of crops and weeds (Fariña et al., 2019), while TICV was shown to be infective in 22 species of weeds that may serve as reservoir of the virus (Alfaro-Fernández et al., 2008). Lastly, another crinivirus that was first reported to infect tomato in China is lettuce chlorosis virus; however, its economic impact has not yet been assessed (Zhang et al., 2017).

### Rhabdoviruses

Rhabdoviruses (*Rhabdoviridae*) can propagate inside either a leafhopper, an aphid, or mite vector or a plant. Rhabdoviruses are composed of large bacilliform virions encapsulating an (−)ssRNA genome of around 13–15 kbp. There are currently three known species of rhabdoviruses that infect or are associated with tomato: the newly discovered TYMaV in genus *Cytorhabdovirus* (Xu et al., 2017) and, in genus *Alphanucleorhabdovirus*, the eggplant mottled dwarf virus (Mavrič et al., 2006) and the recently discovered PhCMoV. PhCMoV was first detected in *Physostegia* sp. sampled in 2014 in Austria (Menzel et al., 2018) and was recently detected in Germany from two tomato samples 12 years apart (2003 and 2015) that showed typical fruit marbling and discoloration symptoms (Gaafar et al., 2018). Within this period, PhCMoV was also detected in tomato samples showing leaf and fruit mottling and uneven ripening of fruits collected in 2012 at three locations in Serbia (Vučurović et al., 2021), indicating extended presence of the virus in the country. Findings of PhCMoV in three different countries in two different plant species in recent years could indicate the emerging status, and the presence of this virus needs to be assessed in the future, to determine its potential impact on tomato production.

### *Secoviridae*: Nepoviruses and Torradoviruses

Family *Secoviridae* contains insect or nematode transmitted viruses, nine of which are known to infect tomato. These viruses have mono- or bipartite 9- to 13.7-kbp-long (+)ssRNA genomes encapsidated in an icosahedral virion (Thompson et al., 2017). Several viruses of this group were reported in recent years, namely, tomato black ring virus (TBRV) from genus *Nepovirus* and ToTV and tomato marchitez virus (ToMarV), both from genus *Torradovirus.* TBRV was recently reported infecting tomatoes in Poland showing chlorotic and/or necrotic ringspots and was found to be associated with eight satellite RNAs, but no assessment about economic losses were made (Rymelska et al., 2013; Zarzyñska-Nowak et al., 2020). Moreover, ToTV is reported to spread globally since its discovery in 2007 in tomatoes from Spain showing systemic necrosis or burnt-like symptoms (Verbeek et al., 2007). ToTV was recently reported to be present in South Africa (Moodley et al., 2016, 2020) and Colombia (Verbeek and Dullemans, 2012), which are first reports of the virus in the Afrotropic and Neotropic ecoregions. ToTV was also reported in tomato-growing areas in the Mediterranean basin (e.g., Spain and Italy), in Poland, Hungary, and Australia (Hanssen et al., 2010a; Hanssen and Lapidot, 2012) and recently in Serbia (Vučurović et al., 2021). On the contrary, since the discovery of ToMarV in tomatoes from Mexico showing severe leaf necrosis and necrotic rings on fruits in 2007 (Verbeek et al., 2008), natural infection of ToMarV was only reported in pepper in Mexico (Camacho-Beltrán et al., 2015).

### Other Known Viruses Associated With Tomato

Several other tomato viruses were recently reported worldwide. Necrotic foliar symptoms in greenhouse grown tomatoes in Poland were associated with an alphanecrovirus (*Tombusviridae*) named olive latent virus 1 (OLV1); however, its economic impact has not been assessed (Borodynko et al., 2010). Aside from its original host, olive, OLV1 was also reported to infect citrus and tulip (Varanda et al., 2014).

In 2015, TMaV, classified in the family *Iflaviridae*, which is currently known to contain members that infect arthropod hosts (Valles et al., 2017), was reported to be associated with tomato (Saqib et al., 2015). The virus was discovered in a single asymptomatic tomato sample in which no presence of a probable arthropod pest was observed under the microscope and in BLAST search of the assembled contigs from small RNA sequencing. Among the artificially inoculated plants, TMaV was detected in non-inoculated leaves, reported to cause symptomless infection in *S. lycopersicum* and *S. melongena*, but showed mild symptomatic infection in *Capsicum annum* (Saqib et al., 2015). Further research is needed to ascertain the role of TMaV in tomato and also to investigate its possible replication in arthropod hosts.

In Brazil, tomato blistering mosaic virus (ToBMV) (*Tymoviridae*) was discovered in field tomatoes that exhibited severe leaf mosaic and blistering symptoms (De Oliveira et al., 2013). Later, analyses confirmed the presence of ToBMV in the tobacco sample from 1986 (Melo et al., 2014). ToBMV was also reported in tomatoes from Argentina (Ferrand et al., 2016) and in a weed relative of tomato, *Solanum violaefolium* (Blawid et al., 2016). These studies may imply possible emergent status of ToBMV in the Neotropics and further investigations are therefore needed.

Southern tomato virus (STV) (family *Amalgaviridae*, genus *Amalgavirus*), a double-stranded (ds) RNA virus, was first detected in tomato plants in United States and Mexico exhibiting stunting of the growing tips, fruit discoloration, and reduced fruit size (Sabanadzovic et al., 2009). Since then, it was also reported in several European countries (Iacono et al., 2015; Verbeek et al., 2015; Pecman et al., 2018; Gaafar et al., 2019; HortDaily, 2019; WUR, 2019), Bangladesh (Padmanabhan et al., 2015c), China (Padmanabhan et al., 2015b), and South Korea (Oh et al., 2018), and so far only in tomato plants. STV co-infections with other viruses are frequent, as well as detections of STV in asymptomatic tomatoes (Alcalá-Briseño et al., 2017). STV virions were not yet observed (Tzanetakis et al., 2020), and the epidemiology of STV remains unclear, as it is only known to be vertically transmitted through seeds at high rates (70–90%) (Sabanadzovic et al., 2009). A recent study showed that STV infection alone did not induce symptoms and cellular ultrastructure changes; however, it modified expression of certain microRNAs (Elvira-González et al., 2020).

### Pospiviroids

Viroids are naked, non-coding, circular (+)ssRNA molecules, around 246–401 bp long, and are the smallest entities known to infect any living organisms. They are known to infect only plants (Kovalskaya and Hammond, 2014). They can induce disease symptoms, resulting in significant economic damages in different crops and ornamentals (Navarro et al., 2012). Viroid infection in tomatoes results in growth reduction or stunting accompanied by leaf distortion or epinasty, although symptoms vary greatly among viroid strains and tomato cultivars (Mackie et al., 2019). Aside from tomato and other crops and ornamentals (van Brunschot et al., 2014a), pospiviroids were also found to be harbored in several weed species, which were reported as their natural reservoir (Van Bogaert et al., 2015; Mackie et al., 2016). Majority of viroids are transmitted mechanically through infected tools and seldom through seeds or pollen; however, the most efficient transmission of viroids is through vegetative propagation of infected material (Flores et al., 2005). Viroids were reported to cause significant losses in tomato production in a few countries over the last decade. Out of the nine recognized pospiviroids (*Pospiviroidae*), eight are known to infect tomato. Among these viroids, potato spindle tuber viroid (PSTVd) and tomato chlorotic dwarf viroid (TCDVd) are the most prevalent and economically important (Verhoeven et al., 2010; van Brunschot et al., 2014b). Several introductions of viroids were reported recently, such as in Italy and Norway, where phytosanitary authorities were able to eradicate PSTVd and TCDVd upon the detection in established tomato crops (Navarro et al., 2009; Fox et al., 2013). In order to prevent introduction of viroids, all imported lots of tomato and pepper seeds are tested in Australia (Constable et al., 2019). Since testing of seeds is needed to prevent the introduction of pospiviroids into new areas, many efforts have already been done to optimize diagnostic protocols that enable their detection at low concentration, which is expected in case of seed infestation (Mehle et al., 2017).

## Conclusion and Future Prospects

New discoveries and studies on emergence of tomato viruses in the recent decade contributed to our understanding of the tomato virome, its diversity, virus ecology, and epidemiology. Tomato is likely associated with the highest number of viruses and viroids known for any plant species to date, currently amounting to at least 312 known species. Because of the absence of resistant varieties for some viruses, global spread of insect vectors, emergence of RB viral strains, increased global movement of plant materials, such as seeds, and high stability and environmental persistence of some viruses, viral diseases in tomato cause massive yield and economic losses. Begomoviruses and associated satellite viruses represent the most numerous group of tomato viruses. ToLCNDV and TYLCV are two of the most important begomoviruses, with ToLCNDV currently prevailing as an emergent and damaging species worldwide (Zaidi et al., 2017; Prasad et al., 2020). On the other hand, a diverse array of RNA viruses cause high economic losses in tomato globally. The most striking example is the recent emergence of ToBRFV that quickly caused outbreaks in several regions all around the world, within just few years after its discovery. According to our review, 45 novel virus species have been discovered in tomato in the past decade and several known viruses have been associated with tomato for the first time. The discovery and first detections of viruses in tomato are increasing in recent years also because of the prevalent use of HTS as a generic virus detection technique. This faster discovery rate is often not paralleled with virus characterization studies, biological or ecological, for newly discovered tomato viruses. In-depth characterization studies are mostly performed for viruses with high phytosanitary and economic importance. At the agroecological scale, the first extensive HTS-based tomato virome studies were conducted in China (Xu et al., 2017) and France (Ma et al., 2020), the latter also addressing the link between the virome of tomato and wild plants. In addition, by the use of HTS, viable tomato-infecting tobamoviruses were detected in the environment outside their hosts, specifically in untreated and treated wastewater (Bačnik et al., 2020).

The increasing knowledge on the virome of tomato, the environment, and the surrounding wild plants contributes to better understanding of virus disease emergence and epidemiology of viruses associated with tomato. Moreover, an increase in viral sequence information in databases can enable more rapid development of targeted diagnostic tests. A tailored approach in studies of plant virus diseases is needed because each crop species or family has its distinctive virome (McLeish et al., 2020b). At the agroecological scale, a holistic view of the virome would include viruses in crops, wild plants, vectors, or in the surrounding environment. This is important to consider for tomato that is associated with a very high number of virus species, which are transmitted by numerous vectors and harbored in wild plants and some remain stable in the environment. HTS-based viromics approach enables collection of ecosystem-level genomic data in extended spatiotemporal scales (McLeish et al., 2020a, b). Furthermore, the use of long-read (or single molecule) portable sequencing platforms, such as Oxford Nanopore Technologies MinIon, is expected to bring HTS even closer to the end-users (e.g., small research and diagnostics laboratories) and contribute to new viral discoveries in plants, including tomato (Bronzato Badial et al., 2018; Boykin et al., 2019; Chalupowicz et al., 2019). Approaches such as virome network analysis can finally be used to, e.g., associate viromes of the crops to that of the insect vectors or wild plants and can consider biogeographical features of the cropping area (Alcalá-Briseño et al., 2020). With such analyses, new insights can be generated that could be useful in possible forecasting and eventual prevention of virus disease emergence and outbreaks in the future (Garrett et al., 2018; McLeish et al., 2020a).

## Author Contributions

MPSR and DK conceptualized the review topic. MPSR did the data mining and analysis and wrote the first draft of the manuscript, including the table, figures, and Supplementary Material. DK contributed in making and editing the table and figures. DK and AV edited initial drafts of the manuscript. DK, AV, NM, and MR edited the final draft of the manuscript. All authors significantly contributed to the writing and editing of the manuscript.

## Conflict of Interest

The authors declare that the research was conducted in the absence of any commercial or financial relationships that could be construed as a potential conflict of interest.

## Supplementary Material

Supplementary Table 1 Meta-analysis of the extent of characterization of novel viruses discovered in tomato from 2011 to 2020.

Supplementary Table 2 Summary list of all viruses with tomato indicated as host based on review of literature and different databases.
